# Transcriptome and Proteome Alternation With Resistance to *Bacillus thuringiensis* Cry1Ah Toxin in *Ostrinia furnacalis*

**DOI:** 10.3389/fphys.2019.00027

**Published:** 2019-02-01

**Authors:** Muhammad Zeeshan Shabbir, Tiantao Zhang, Zhenying Wang, Kanglai He

**Affiliations:** State Key Laboratory for Biology of Plant Diseases and Insect Pests, Institute of Plant Protection, Chinese Academy of Agricultural Sciences, Beijing, China

**Keywords:** *Ostrinia furnacalis*, *Bacillus thuringiensis*, Cry1Ah toxin, qRT-PCR, RNA-Seq, iTRAQ

## Abstract

**Background:** Asian corn borer (ACB), *Ostrinia furnacalis* can develop resistance to transgenic *Bacillus thuringiensis* (Bt) maize expressing Cry1Ah-toxin. However, the mechanisms that regulate the resistance of ACB to Cry1Ah-toxin are unknown.

**Objective:** In order to understand the molecular basis of the Cry1Ah-toxin resistance in ACB, “omics” analyses were performed to examine the difference between Cry1Ah-resistant (ACB-AhR) and susceptible (ACB-BtS) strains of ACB at both transcriptional and translational levels.

**Results:** A total of 7,007 differentially expressed genes (DEGs) and 182 differentially expressed proteins (DEPs) were identified between ACB-AhR and ACB-BtS and 90 genes had simultaneous transcription and translation profiles. Down-regulated genes associated with Cry1Ah resistance included aminopeptidase N, ABCC3, DIMBOA-induced cytochrome P450, alkaline phosphatase, glutathione *S*-transferase, cadherin-like protein, and V-ATPase. Whereas, anti-stress genes, such as heat shock protein 70 and carboxylesterase were up-regulated in ACB-AhR, displaying that a higher proportion of genes/proteins related to resistance was down-regulated compared to up-regulated. The Kyoto encyclopedia of genes and genomes (KEGG) analysis mapped 578 and 29 DEGs and DEPs, to 27 and 10 pathways, respectively (*P* < 0.05). Furthermore, real-time quantitative (qRT-PCR) results based on relative expression levels of randomly selected genes confirmed the “omics” response.

**Conclusion:** Despite the previous studies, this is the first combination of a study using RNA-Seq and iTRAQ approaches on Cry1Ah-toxin binding, which led to the identification of longer length of unigenes in ACB. The DEGs and DEPs results are valuable for further clarifying Cry1Ah-mediated resistance.

## Introduction

Maize (*Zea mays* L.) is the main crop in terms of production and planting area (Wang et al., 2014). Asian corn borer (ACB), *Ostrinia furnacalis* (Guenée), is an economically important pest of maize causing 20–80% yield losses (Nicolas et al., 2013) by attacking fresh whorl leaves, silks, ears, and cobs, finally leading to devastation by boring into the stalk, ear shanks and cobs of corn (He et al., 2003). The potential of ACB having adaptation to many host crops and higher fecundity are the key factors in developing Bt resistance (Zhang et al., 2014). Genetically modified crops produced by Bt are effective in controlling this endemic pest of maize, likewise, the transgenic Bt maize, Cry1Ac, Cry1Ab, Cry1Ie, and Cry1Ah which express transgenic insecticidal proteins are assumed to show effectiveness against infestation of ACB (Zhang et al., 2013; Shabbir et al., 2018). Although Bt transgenic crops are likely to hold great promise to improve insect pest management, the efficacy of Bt maize can be reduced by the evolution of target insect resistance. The increased occurrence of functional resistance in the pest populations is causing hazardous loss to the continuing success of Cry proteins (Tabashnik et al., 2003). Previously, evolution of potential resistance to various Bt toxins Cry1Ac, Cry1Ie, and Cry1F has been observed in ACB in laboratory selection (He et al., 2003; Wang et al., 2016), and now one ACB-AhR strain had developed resistance to Cry1Ah, and readily consumed Cry1Ah-Bt maize (Shabbir et al., 2018).

However, complete recognition of the mechanism of Bt resistance is essential to delay the resistance evolution in target insect pest. Currently, two different hypotheses for modes of actions are directed for Cry toxin: the pore formation model and signal transduction model (Soberón et al., 2009). The pore formation model has been reported to propose that reduction of Bt toxins in toxin binding sites in brush border membrane vesicles (BBMVs) of insect midgut is the major factor of the evolution of resistance in target insect pests (Daniel et al., 2002). After the crystalline inclusions, toxins are ingested and solubilized in the gut to the protoxin, which is cleaved by midgut proteases and binds to activated toxins (Bravo and Soberon, 2008; Soberón et al., 2009). The interaction of toxins with cadherin enables additional proteolytic cleavages that prompt the toxin oligomerization. Subsequently, these oligomers bind to secondary receptors, aminopeptidase N (APN) and alkaline phosphatase (ALP), as they have a larger affinity to bind these proteins as compared to the monomeric toxin. After binding, these oligomers insert into the membrane and create pores which make it more permeable. Finally, these pores cause osmotic shock in the membrane, ultimately leading to the death of cells (Soberón et al., 2009). According to the signal transduction model, the binding of Cry1A to cadherin is supposed to activate a cascade pathway involved in the stimulation of a G protein and adenylate cyclase to increase cAMP, causing activation of protein kinase A, and finally death of the cell (Zhang et al., 2006). Previously, several studies have reported binding receptors, including cadherin protein (Xu et al., 2005), APN (Tiewsiri and Wang, 2011), ALP (Jurat-Fuentes and Adang, 2007), membrane glycolipids (Griffitts and Aroian, 2005), and ABCC2 of ABC transporters (Gahan et al., 2010; Baxter et al., 2011). The differences in the sequences of amino acids and expression of mRNA of four APN genes have been observed between ACB-AbR and ACB-BtS strains (Xu et al., 2014). In addition, V-type ATPase and HSP 70 kDa proteins had been documented as Bt binding proteins in ACB using a proteomic approach (Xu et al., 2013). However, the studies describing the Bt resistance mechanism are still limited in ACB.

Gene expression analysis is extensively used for studying regulatory mechanisms that control cellular processes in plants, animal, and microbes. Recent advancement in high-throughput RNA sequencing (RNA-seq) technology and isobaric tags for relative and absolute quantification (iTRAQ) gene expression based on next generation sequencing technology significantly has upgraded transcriptome analysis (Wang et al., 2010; Chen et al., 2011). In the present study, we compared midgut tissues of ACB-AhR and ACB-BtS strains at transcriptome (RNA-seq) and proteome (iTRAQ) level to determine the molecular mechanism of Bt Cry1Ah resistance in ACB. The differentially expressed genes (DEGs) and differentially expressed proteins (DEPs) were further validated by quantitative real-time qRT-PCR analysis. These approaches are valuable for the understanding of systemic differences between susceptible and Bt resistant genotypes, and to identify the genes/proteins that might be involved in conferring resistance to Cry1Ah-toxin.

## Materials and Methods

### Insects

The susceptible strain (ACB-BtS) and the Cry1Ah resistant strain (ACB-AhR), as reported previously (Shabbir et al., 2018), were used in the study. In our previous study, ACB-AhR had developed 200-fold resistance to Cry1Ah after 48 generations of selection (Shabbir et al., 2018). However, in the present study, the ACB-AhR was selected to detect the Cry1Ah resistance-relative genes in ACB. Four to five individual larvae from fifth instar larvae were collected as one biological replicate for both ACB-BtS and ACB-AhR. Three biological replicates for each sample were collected and processed independently. Three replicates were used in gene expression profile analysis, and Illumina sequencing, as well as three biological replicates which were used for the qRT-PCR analysis. All samples were stored at -80°C until assayed.

### Library Preparation for Transcriptome Sequencing

A total amount of 1.5 μg RNA from the fifth instars larvae was used as input material for RNA sample preparation for each of the ACB-AhR and ACB-BtS strains. Sequences libraries were generated using NEBNext^®^ Ultra^TM^ RNA Library Prep Kit for Illumina (NEB, United States) according to manufacturer’s instructions and index codes were added to attribute sequences to each sample. The mRNA was purified from total RNA using poly-T oligo-attached magnetic beads and broken into short fragments using divalent cations under elevated temperature in NEBNext First Strand Synthesis Reaction Buffer (5X). First-strand cDNA was synthesized using random hexamer primer and M-MuLV Reverse Transcriptase (RNase H^-^). Second-strand cDNA was subsequently performed using DNA polymerase I and RNase H. Remaining overhangs were converted into blunt ends via exonuclease/polymerase activities. After adenylation of 3′ ends of DNA fragments, NEBNext Adaptor with a hairpin loop structure was ligated to prepare for hybridization. In order to select cDNA fragments of preferentially 250–300 bp in length, the library fragments were purified with AMPure XP system (Beckman Coulter, Beverly, MA, United States). Then before PCR, 3 μl USER Enzyme (NEB, United States) was used with size-selected, adopter-ligand cDNA at 37°C for 15 min followed by 5 min at 95°C. PCR was performed with Phusion High-Fidelity DNA polymerase, Universal PCR primers, and Index (X) Primer. Finally, PCR products were purified (AMPure XP system) and library quality was assessed on the Agilent Bioanalyzer 2100 system. The RNA-seq data has been submitted to SRA database and the accession ID is PRJNA508227.

### Assembly and Functional Gene Annotation

The reads containing ploy-N (<10%), and low quality reads (*q* < 20) were removed from raw data. Q20, Q30, GC-content and sequence duplication level of the clean data were also assessed based on high quality clean data. Subsequently, the clean reads were accomplished using Trinity software (Grabherr et al., 2013). Gene functional annotation sequences were searched using BLAST against NCBI NR database was searched using BLAST against NCBI NR database^1^ with a cut-off E-value of 10^-5^. Functional gene annotations were collected for transcript sequences ≥150 bp using Blast2GO (Conesa et al., 2005). DEGs were calculated in FPKM (fragments per kilobase pair of exon model per million fragments mapped) for comparing the expression of up- or down-regulated transcripts in two groups. BLASTx algorithm was used to assign gene ontology (GO) terms from the GO database^2^ and the DEGs were assigned into different pathways by the Kyoto encyclopedia of genes and genomes (KEGG) pathways databases.

### Screening of Differentially Expressed Genes Between ACB-AhR and ACB-BtS

The mapped reads of ACB-AhR and ACB-BtS groups were assembled using the DESeq (2010) R package (1.10.1). DESeq fetches statistical routines to regulate differential expression in digital gene expression data using a model based on the negative binomial distribution. The resulting *P-*values were adjusted using the *q*-value. Genes with an adjusted *P-*value <0.05 found by DESeq were assigned as differentially expressed. Then, the FPKM value between the biological replications was analyzed for each gene. The significance of digital gene expression profiles was analyzed as described previously (Audic and Claverie, 1997). The fold change of each gene was then calculated by the formula of log_2_ (ACB-AhR_FPKM/ACB-BtS_FPKM). False discovery rate (FDR) method was used to determine the threshold of *P-*value in differential gene expression tests. “FDR” ≤ 0.05 and the absolute value of log_2_-ratio ≥ 1” was the threshold to evaluate the significance level of differentiated gene expression for comparing the gene expression between two strains of ACB.

### Protein Quantification and Database Search Using iTRAQ Labeling

The midgut tissues of ACB-AhR and ACB-BtS samples were individually milled in liquid nitrogen then put into 1 ml of lysis buffer (50 mM Tris buffer, 8 M urea, 1% SDS, pH 8), and ultrasonic was used to extract the protein. The lysis solution was centrifuged at 4°C, 12,000 × *g* for 15 min to collect the supernatant, then four volumes of precooling acetone (include 10 mM DTT) was added to a sample extract, and samples were placed at 20°C for 2 h. It was centrifuged again, and the pellet was collected to wash twice with cold acetone. Finally, the precipitation was dissolved by the dissolution buffer containing Tris-base (pH 8) 8M Urea solution. The protein was determined by using the Bradford method and analyzed on the SDS-PAGE gel. After 100 ml protein from each sample was digested with trypsin gold (Promega, Madison, WI, United States) at 37°C for 16 h, and the resultant peptides were dried by vacuum centrifugation. The peptides were reconstituted in 20 μl of 0.5 M TEAB (pH 8.5) and processed according to the manufacturer’s protocol for 8-plex iTRAQ (AB Sciex, Foster City, CA, United States) (Noirel et al., 2011). Then, pooled mixtures of iTRAQ-labeled peptides were fractionated by XBridge BEHC18 column BEH C18 4.6 × 250 mm, 5 μm, (Waters, Milford, MA, United States) on a Rigol L3000 HPLC operating at 1 ml/min. Mobile phases A (2% acetonitrile, 20 mM NH_4_FA, adjusted pH to 10.0 using NH_3_⋅H_2_O) and B (98% acetonitrile, 20 mM NH4FA, adjusted pH to 10.0 using NH_3_⋅H_2_O) were used to develop a gradient elution. Collected fractions were pooled into 15 final fractions and analyzed by Q-Exactive HF-X mass spectrometer (Matrix Science Limited, Washington, DC, United States).

Peptides were identified separately by searching against a specified database Proteome Discoverer 2.2 (PD 2.2, Thermo). A peptide mass tolerance of 10 ppm and fragment mass tolerance of 0.02 Da were acceptable for product ion scans. When the Proteome Discoverer 2.2 software was used to search the database, 5,900 proteins were identified at FDR less than 1.0%. Proteins comprising of similar peptides and could not be distinguished based on MS/MS analysis were grouped separately as protein groups. To analyze the differential expression ratios, all identified peptides from a protein were used to find an average protein ratio relative to the control label (i.e., fold change). Mann–Whitney test was used to analyze the differential expression of proteins between ACB-AhR and ACB-BtS larvae midgut and the significant ratios, defined as *P* < 0.05 and | log_2_FC| > ^∗^(ratio > ^∗^ or ratio < ^∗^[fold change, FC]), were used to screen the DEPs.

### GO Classification of Differentially Expressed Genes and Proteins Pathway Enrichment Analysis

Functional annotation of the genes and proteins which were identified in ACB midgut sample was implemented using GOseq R packages based Wallenius non-central hyper-geometric distribution (Young et al., 2010), an integrated GO annotation and mining tool that assigns gene ontology through BLAST searches against nucleotide and protein databases. GO functional significance enrichment analysis gives GO functional entries that are significantly enriched in DEGs compared to the genomic background. The analysis first maps DEGs to each term in the Gene Ontology database (see footnote 2), calculate the number of genes for each term, and then find differences in expression compared to the entire genomic background and then used a hypergeometric test to find significantly enriched GO terms for DEGs compared to the ACB transcriptome/proteome background. In order to better study the function of differential genes, we not only performed enrichment analysis (GO enrichment, KEGG enrichment) for all the differential genes in each combination but also separated differential genes in each combination according to up- or down-regulation. The differential expression of the genes was determined by performing independent alignments of short reads count obtained from analysis of gene expression levels. For samples with biological replicates, the analysis was performed using DESeq (Anders and Huber, 2010), and the screening threshold was *p*_adj_ < 0.05. The *P-*value was checked by using the following formula:

$$P=1-\sum_{i=0}^{m-1}\frac{\left(\begin{array}{c} M\\ i \end{array}\right)\left(\begin{array}{c} N-M\\ n-i \end{array}\right)}{\left(\begin{array}{c} N\\ n \end{array}\right)}$$

(1) *N* is the number of genes with pathway annotation in all genes. (2) *n* is the number of DEGs in *N*. (3) *M* is the number of genes annotated as a particular pathway in all genes. (4) *m* is the number of DEGs annotated as a specific pathway. Pathway with *FDR* ≤ 0.05 was defined as a pathway that was significantly enriched in DEGs or proteins. All identified transcripts and proteins were mapped to a pathway in the KEGG database. Significantly enriched metabolic pathways containing DEGs and DEPs were determined using the same formula as in GO analysis. Here *N* means the number of all the genes/proteins with KEGG annotation, *n* represents the number of DEGs or DEPs in *N, M* is the number of all genes or proteins annotated to specific pathways, and *m* is the number of DEGs or DEPs in *M*.

### Relationship Between RNA-Seq and iTRAQ

To evaluate the expression level of genes and proteins in ACB-AhR and ACB-BtS, the relationship between transcriptomic and proteomics levels was evaluated. The mRNA information obtained from the transcriptome was integrated with the DEPs information identified by the proteome and was searched for the expression patterns of corresponding genes (*P* < 0.05). The significance of the overlapping between the identified transcripts and proteins was determined using Pearson’s chi-square test with Yates’ continuity correction (Song et al., 2012).

### RT-qPCR for Expression Analysis

The genes related to resistance selected from transcriptomic and proteomic analysis were verified using qRT-PCR. Total RNA was prepared from different tissues of ACB-AhR and ACB-BtS strains, with three technical replicates performed for each of three biological replicates. cDNAs were synthesized using the One-Step gDNA Removal and cDNA Synthesis SuperMix (TransGen Biotech Co., Ltd., Beijing, China) following the kit manual. *β-actin* was used as a reference gene (accession number-EU585777.1), and it was used to select the cDNA templates on the PCR equipment. Primers (Supplementary Table S9) were designed manually or using the Primer 5 tool^3^. Individual qRT-PCR reactions were repeated four times; water was used as the negative control. Before gene quantification, the amplification efficiency between the target gene and the reference gene were checked. qRT-PCR reactions were performed on the Applied Bio System 7500 Real-Time PCR System (Applied Biosystems, Foster City, CA, United States) using SYBR Green (TAKARA Bio Inc., Japan) The cycling program consists of initial incubation at 95°C for 10 min, followed by 40 cycles at 95°C for 15 s, 60°C for 45 s, and a final step at 95°C for 15 s and reactions were performed in a final volume of 25 μl. The threshold cycle (*C*_T_) was collected from each reaction, and the relative expression of normalized data was calculated by the comparative 2^-ΔΔCT^ method (Livak and Schmittgen, 2001; Zhang et al., 2017).

## Results

### RNA-Seq and Sequence Assembly

The results of RNA sequencing from ACB-AhR and ACB-BtS were ranged from 41,703,706 to 62,099,678 (Table 1). The clean sequences per library were ranged from 40,607,798 to 59,909,406 reads. Moreover, GC contents were ranged from 48.01 to 51.02%. The number of the reads ranged from 40.09 to 44.83%, were mapped to the trinity spliced transcriptomes. A total of 73,229 unigenes assembled from cDNA libraries of both resistant and susceptible strains with an average length of 844 bp and N50 length of 1,018 bp (Table 2).

**Table 1:** Summary of reads in Cry1Ah-resistant strain (ACB-AhR) and susceptible strain (ACB-BtS) of *Ostrinia furnacalis* transcriptomes.

Samples	Raw reads	Clean reads	Total mapped	Q30 (%)	GC (%)
ACB-BtS_1	54,874,002	53,134,612	21,299,364 (40.09%)	90.90	50.55
ACB-BtS_2	41,970,856	40,607,798	16,906,344 (41.63%)	91.55	51.02
ACB-BtS_3	54,469,658	52,770,170	21,864,498 (41.43%)	91.50	50.40
ACB-AhR_1	41,703,706	40,498,994	17,492,734 (43.19%)	91.81	48.11
ACB-AhR_2	62,099,678	59,909,406	24,716,016 (41.26%)	91.19	50.71
ACB-AhR_3	47,695,548	46,435,464	20,817,278 (44.83%)	92.06	48.01


**Table 2:** Summary of RNA-seq metrics from *Ostrinia furnacalis* midgut transcriptomes.

Metrics	Number
Number of genes	73,229
^∗^N50	1,018
Maximum length	29,484
Minimum length	201
Average length	844
Total assembled bases	132,050,968


*^∗^N50 is the 50% length of all genes.*

The total numbers of sequences detected by mass spectrometry of ACB proteome were 585,828, which represented 29,314 peptide spectra and 5,900 proteins were matched (Table 3). The total DEPs between ACB-AhR and ACB-BtS were 182.

**Table 3:** Summary of iTRAQ metrics from the Cry1Ah-resistant strain (ACB-AhR) and susceptible strain (ACB-BtS) of *Ostrinia furnacalis* proteomes.

Metrics	Number
Total spectra	585,828
Unique spectra	29,314
Matched proteins	5,900
Differentially expressed proteins	182


### Differentially Expressed Genes Between Cry1Ah-Resistant and Susceptible Strains of ACB

**FIGURE 1:**
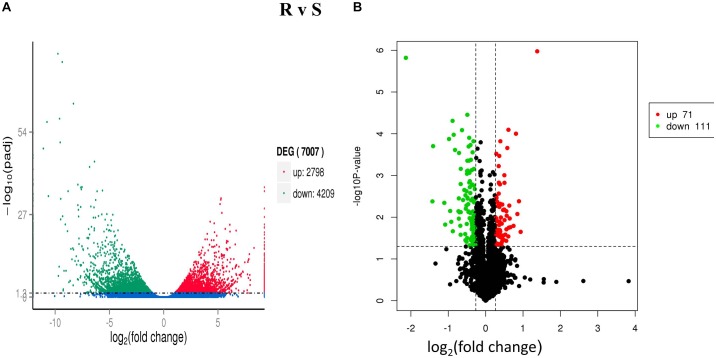
Differentially expressed genes (DEGs) and proteins (DEPs) between Cry1Ah-resistant and susceptible *Ostrinia furnacalis*. **(A)** The distribution of DEGs. The scatter in the figure represents each gene, green genes are down-regulated, red genes are up-regulated, and blue genes are not differentially expressed. **(B)** The distribution of differentially expressed proteins (DEPs). The vertical axis indicates the *P*-value (–log_10_ value), black indicates the protein with no significant difference, red indicates the up-regulated proteins (≥1.2-fold, *P* ≤ 0.05), and green indicates the down-regulated proteins (≤0.8-fold, *P* ≤ 0.05).

A total of 4,209 down-regulated and 2,798 up-regulated genes were differentially expressed (*P* < 0.05 and | log_2-_ratio ≥ 1) (Figure 1A) in both ACB-AhR and ACB-BtS strains. These comparison results revealed that most of the genes were significantly down-regulated compared to up-regulated including APN, ALP, and member of the ABC the transporter family (Supplementary Table S1). Furthermore, genes significantly down-regulated in the high severity in ACB-AhR strain with threshold group (*q*-value <1 and log_2_ (fold-change) ≤-2), several genes were annotated as previously known Bt resistance genes including members of the APN gene family, *apn*3 paralogs and *apn*8, an ABC transporter in subgroup G, *abcg*, and serine protease genes. The up-regulated genes (*q*-value <1 and log_2_ (fold-change) ≥2) were significantly smaller in number for ACB-AhR strain compared to down-regulated genes. The up-regulated genes in ACB-AhR strain included heat shock proteins and carboxylesterase genes (Supplementary Table S1).

**FIGURE 2:**
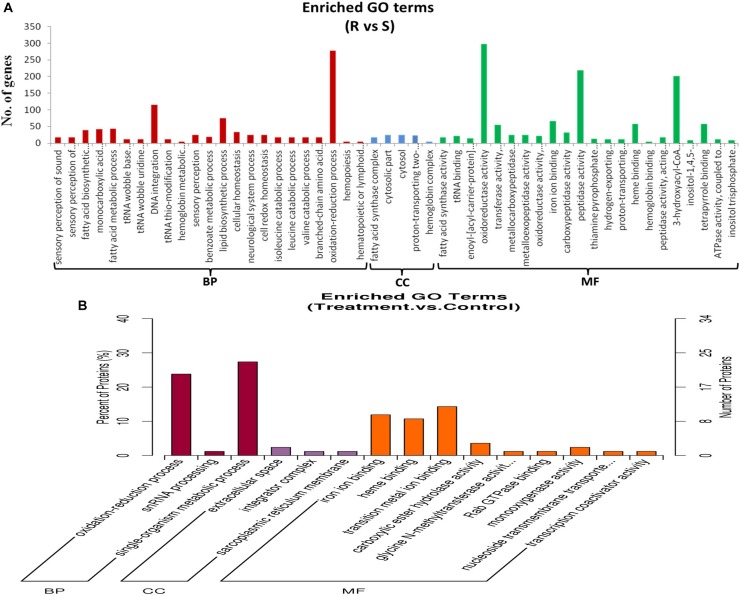
Gene ontological classifications of differentially expressed genes and proteins between ACB-AhR and ACB-BtS. The differentially expressed genes or proteins are grouped into three hierarchically stretched GO terms, biological process, cellular components, and molecular functions. The *Y*-axis indicates the number of genes or proteins in each GO term. **(A)** Differentially expressed genes identified by RNA-seq. **(B)** Differentially expressed proteins identified by iTRAQ.

Supplementary Table S2 shows the GO classification of genes that were differentially expressed between ACB-AhR and ACB-BtS midgut tissues (≥2-fold change, FDR ≤ 0.001). With Blast2Go, 7,007 DEGs were assigned to 51 GO classes (Figure 2A), which cover three domains: biological process, cellular components, and molecular functions. In terms of biological process mostly genes are assigned to oxidation–reduction process and DNA integration. In case of oxidation reduction reaction, 277 DEGs were associated, where 162 were down-regulated and 115 DEGs were up-regulated in ACB-AhR (Supplementary Table S2). In case of cellular components terms, mostly fatty acid synthesis complex, and cytosolic part represented most of the genes. In the molecular function category, oxidoreductase activity, peptidase activity, and dehydrogenase activity were the most abundant (Supplementary Table S2).

**FIGURE 3:**
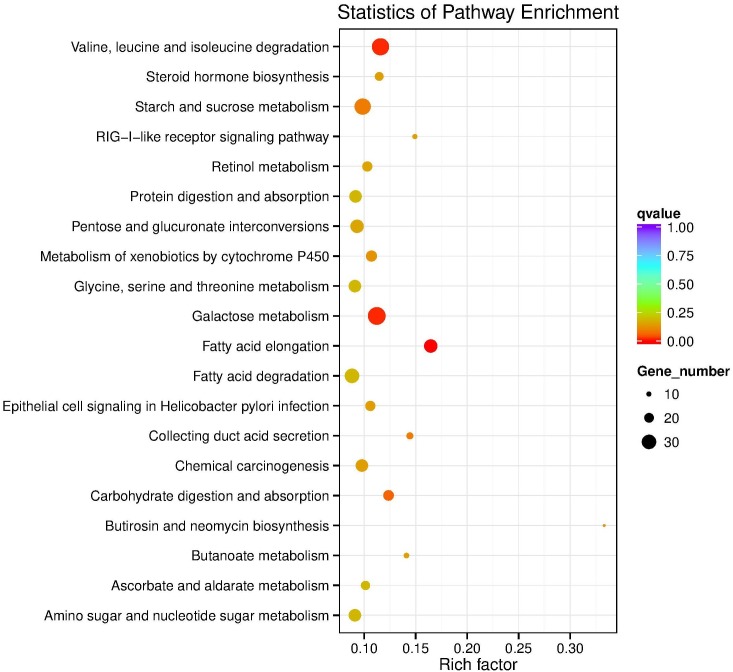
KEGG pathway enrichment scatter plot. The vertical axis represents the path name, and the horizontal axis represents the path factor corresponding to the Rich factor. The size of the *q*-value is represented by the color of the point. The smaller the *q*-value, the closer the color is to the red color. The number of differential genes included in each pathway are expressed by the size of the point. (Top 20 enriched pathways are represented in scatter plot).

In the KEGG database, 27 pathways were substantially enriched (*P* ≤ 0.05), including “Valine, leucine and isoleucine degradation” and “Galactose metabolism” (Figure 3 and Supplementary Table S3). Specifically, 51 genes encoding enzymes involved in fatty acid elongation and metabolism of xenobiotics by cytochrome P450 pathways were highly enriched, including dehydrogenase, glutathione *S*-transferase (GSTs), and nicotinamide adenine dinucleotide phosphate (NADPHs) (Supplementary Table S4). The up-regulated genes included acetyltransferase, dehydrogenase, GST, and carbonyl reductase NADPH. Whereas, down-regulated genes enriched in galactose metabolism pathways, included steroid dehydrogenase and UDP-glucosyltransferase (Supplementary Table S4).

### Cry1Ah-Induced Differentially Expressed Proteins Between ACB-AhR and ACB-BtS Strains

After Cry1Ah-treatment, 182 DEPs (*P* ≤ 0.05) were identified between ACB-AhR and ACB-BtS strains of ACB (Figure 1B). Among them, 111 proteins were down-regulated (≤0.8-fold, *P* ≤ 0.05) and 71 proteins were up-regulated (≥1.2-fold, *P* ≤ 0.05) (Supplementary Table S5). Following in-gel digestion by trypsin, proteins were identified by liquid chromatography-electrospray ionization multistage mass spectrometry (LC-ESI-MS/MS). APN and ABCC proteins which are involved in Bt resistance were down-regulated by -0.45- and -0.51-fold, respectively, in ACB-AhR strain relative to the ACB-BtS strain. Others down-regulated proteins in resistance included trypsin (-1.41-fold), which are considered the main proteases involved in Bt protoxin activation and detoxification, GST (-0.67-fold), and DIMBOA-induced cytochrome P450 (-0.46-fold). The proteins that were up-regulated in Cry1Ah-resistant insects of ACB are fatty acid binding protein 1 (0.41-fold), aldose 1-epimerase (0.50-fold) involved in carbohydrate metabolic process, lipase (0.58-fold), plays an essential role in the digestion, transport and metabolism and UDP-glycosyltransferase (0.42-fold), involved in inactivation and excretion of endogenous and exogenous compounds. Additionally, proteins related to energy regulations, transportation of proteins, oxidation–reduction process, binding, and metabolism were also differentially expressed between ACB-AhR and ACB-BtS strains of ACB (Supplementary Table S5).

**FIGURE 4:**
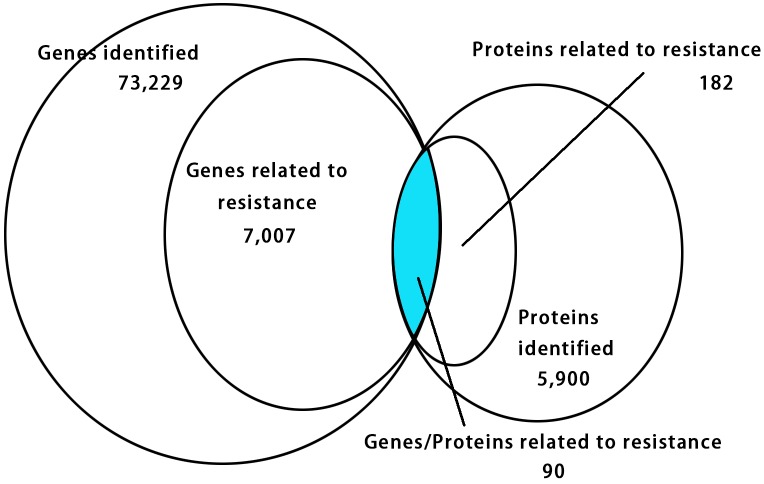
Venn diagram showing relationship between the differentially expressed proteins and genes. The values in each circle represents the quantity of genes or proteins, including identified genes and proteins and genes and proteins related to resistance, respectively, genes/proteins related to resistance together. The cut-off value of log_2_ fold changes for up-regulated and down-regulated gene/protein was +1/–1.

The relationship of correlation between the DEGs and DEPs showed that there were only 90 genes/proteins related to resistance that were either up-regulated or down-regulated identified in RNA-seq and iTRAQ techniques (Figure 4 and Supplementary Table S6). Among them 63 genes/proteins were with same trend and 27 genes/proteins showed opposite trend either up-regulated or down-regulated in both analyses (Supplementary Table S6).

### Gene Ontology and Pathway Enrichment

Among the 182 DEPs, 34 were subcategorized into 15 hierarchically structured GO classes, including 3 biological processes, 3 cellular components, and 9 molecular functions (Figure 2B). Specifically, “oxidation–reduction process” and single-organism metabolic process were highly represented in “Biological process”. While extracellular space was the most common categories in “Cellular components”. Likewise, iron ion binding, heme binding, and transition metal ion binding were the most top categories in “Molecular function”.

**FIGURE 5:**
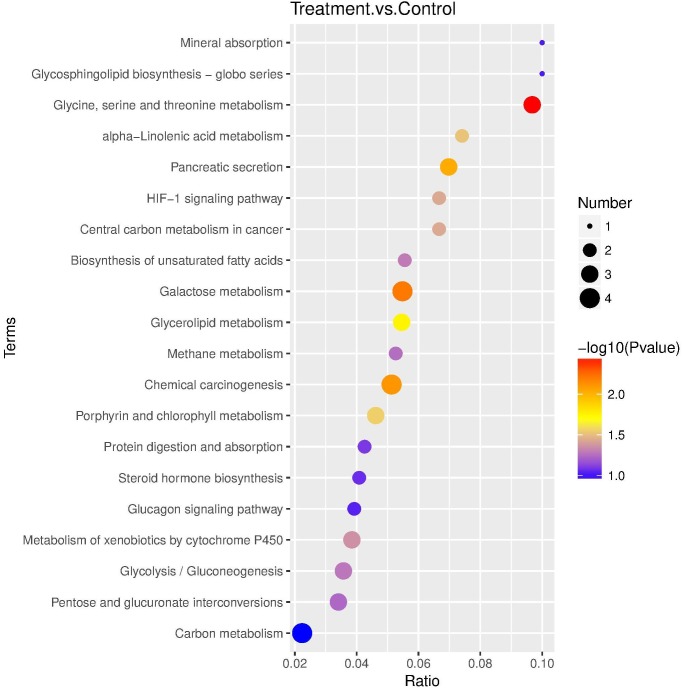
KEGG pathway enrichment bubble plot. The ratio of the number of different proteins in the corresponding pathways to the total number of proteins identified in the graph is greater, indicating the higher difference in protein concentration in the pathway. The size of the dots represents the number of different proteins in the corresponding pathway and the greater the difference in the pathway represents the greater number of proteins.

Fifty-nine DEPs were allocated to reference pathways in KEGG when exposed to Cry1Ah toxin. As a result, 10 pathways were enriched *P* ≤ 0.05, Supplementary Table S7), including “glycine, serine, and threonine metabolism” and “galactose metabolism” which have the lowest *P-*value. The top 20 highly enriched pathways are shown in Figure 5.

Correlation of the enriched pathways for DEGs and DEPs showed that there were four mainly identical pathways related to metabolic process playing a role in resistance, including determining, galactose metabolism, glycerolipid metabolism, metabolism of xenobiotics by cytochrome P450 and glycine, serine, and threonine metabolism (Figure 3, 5). KEGG pathway analysis also revealed that the most enriched peptides, including phosphoglycerate dehydrogenase, *N*-acetylglactosaminidase, NADPH, and UDP-glycosyltransferase were involved in glycine, serine, and threonine metabolism, galactose metabolism, and metabolism of xenobiotics by cytochrome P450 (Supplementary Table S8).

### Validation of Differentially Expressed Genes by qRT-PCR

**FIGURE 6:**
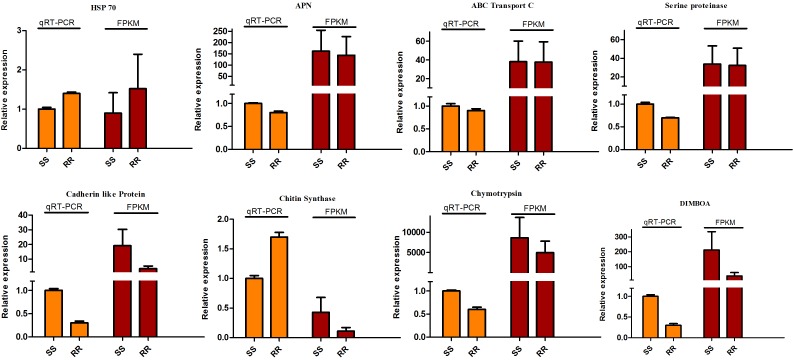
The qRT-PCR analysis of differentially expressed genes to confirm expression patterns indicated by sequencing. The DEGs were randomly selected for qRT-PCR. Three technical replicates were performed for each of the three biological replicates. The height of each bar chart represents the mean average of sample-specific 2^-ΔΔCt^ values.

According to fold-change calculations by qRT-PCR analyses, the results supported the differentially expressed on gene level. All the tested genes were in the same trend with the omics results except the chitin synthase which presented the down-regulation in Cry1Ah-resistant (ACB-AhR) strain compared to susceptible strain (ACB-BtS). However, the higher expression level was observed in ACB-AhR by qRT-PCR analysis (Figure 6). Most of the selected genes were down-regulated in ACB-AhR; only HSP 70 showed higher expression in ACB-AhR compared to ACB-BtS (Figure 6).

## Discussion

Insect resistance to *Bacillus thuringiensis* (Bt) is a significant threat to the enduring success of most extensively used genetically modified crops (Tabashnik et al., 2003, 2013). To counter the threat of resistance, it is important to understand the molecular mechanism of resistance of ACB to Bt toxins. In this study, ACB-AhR and ACB-BtS were sequenced for the transcriptomics and proteomics analyses, and we obtained a total of 73,229 genes with an average length of 844 bp from the transcriptome analysis. The average length of the genes was longer than those observed in ACB (Xu et al., 2015; Zhang et al., 2016; Cui et al., 2017), and *Plutella xylostella* (Lin et al., 2013). The genes length may be correlated to sequence techniques and the application of assembly tools. Mostly, assembled genes were not significantly matched with available databases due to their short sequences or because they characterized significantly novel genes. Comparatively, a low number of the genes had been annotated previously as compared to our findings. Therefore our Illumina sequencing and analysis described improvements over earlier studies (Xiang et al., 2010; Li et al., 2013).

Particularly, comparative analysis of midgut transcripts and proteins between ACB-AhR and ACB-BtS strains discovered a distinctive set of genes/proteins differentially expressed. Both transcriptomic and proteomic sequences showed more down-regulation of genes/proteins than up-regulations in ACB-AhR strain (Figure 1). Specifically, our results are in agreement with a previous transcriptomic analysis showing down-regulation of genes in resistant strains using a digital gene expression tag profiling (DGETP) approach (Paris et al., 2012; Tetreau et al., 2012). Similarly, significant alteration of the ACB transcriptome was observed in a Cry1Ab resistant strain (ACB-AbR) including, 3,157 genes being down-regulated and 636 were up-regulated after exposure to Cry1Ab toxin (Xu et al., 2015). Moreover, in a previous study, an analysis of DEGs directed that 1,026 DEGs were down-regulated and 189 were up-regulated, expressed between resistant and susceptible strains of *P. xylostella* (Lin et al., 2013). However, a study of transcriptome response to Cry1Ac toxin indicated more up-regulated genes as compared to down-regulated genes in a Cry1Ac-resistant strain of *P. xylostella* (Lei et al., 2014). The observation of different trends among experiments was possibly due to the technical differences and the variations in the materials examined, as a whole body of target insects at various developmental stages was used in susceptible and Cry1Ab-resistant strains of *P. xylostella* (Lin et al., 2013). However, midgut tissue was used from Cry1Ac-resistant and susceptible strains of *P. xylostella* (Lei et al., 2014). These results suggest that mechanisms of resistance to Cry toxins can be conferred by deficient activation of protoxins or reduced binding of toxins to the membrane (Griffitts and Aroian, 2005).

A correlation analysis of DEGs and DEPs from the larval midgut displayed the same trend of a subset of genes and proteins (Supplementary Table S6). Genes including ABC transporter C2, DIMBOA-induced cytochrome P450, cadherin-like protein, and chymotrypsin-like serine protease were down-regulated, whereas aldehyde dehydrogenase and *N*-acetylglactosaminidase were up-regulated at both transcriptional and translational levels (Supplementary Table S6). Likewise, physiologically similar responses were documented in *Sarcophaga crassipalpis, Drosophila melanogaster*, and *Caenorhabditis elegans* transcriptomes (Ragland et al., 2010). However, we found some genes with the opposite trends, like the trypsin-like serine protease and NADH dehydrogenase were up-regulated at the transcriptional level and down-regulated at the translational level. This effect could be attributed to the difference in expression time (Ragland et al., 2010). Moreover, expression profiles of mRNA and protein levels do not always correlate (Nie et al., 2006), and differences in directional changes between proteomic and transcriptome are possibly due to the single sampling time-point and changes in protein versus genes *in vivo* are rarely studied (Popesku et al., 2010). Similarly, the difference between differentially expressed transcripts and proteins will most likely be the normal rather than exception, without a fully sequenced ACB genome.

In the present study, several transcripts which are down-regulated in the ACB-AhR strain were previously documented as important candidate Bt resistance genes/proteins or other genes involved in insecticide resistance in numerous insects including APN, ABCC3, V-ATPase, trypsin-like serine protease, DIMBOA-induced cytochrome P450, ALP, GST, chymotrypsin-like serine protease family members and chitin synthase (Supplementary Table S1). The significant correlation between transcriptome/proteomic and qRT-PCR results further verified the gene expression data, providing assurance in the reliability of our data (Figure 6). Different isoforms of APNs and CAD together with ALP have been reported as Cry toxin receptors (Pigott and Ellar, 2007). The same phenomenon of down-regulation of cadherin as a Cry toxin receptor was previously described in ACB-AbR, and AcR strains in both microarrays and qRT-PCR results (Zhang et al., 2017), supporting the results of a prior study which indicated the down-regulation of *Ofcad* gene in Cry1Ac-resistant strain (Jin et al., 2014). Down-regulation of APN transcripts in resistant strains has been shown to be involved in the Bt mode of action and mechanisms of the resistance are reported through proteomics and molecular analyses to different Cry toxins (Nanoth et al., 2015; Zhang et al., 2017). Interestingly, we also found dozens of genes annotated to APN were over-expressed in ACB-AhR strain. GO, and up-regulation (2.47 to 5.65-times) of APN1 (ABQ51393.1), APN2 (ACF34999.1), APN3 (AEO12689.1), and APN4 (ACF34998.2) of APN in Cry1Ab resistance in ACB-AbR (Xu et al., 2015). It was also reported that APN encoded by the Unigenes59183-mk was significantly up-regulated in a Cry1Ac-resistant strain of *P. xylostella* (Lei et al., 2014), and AAEL012774 annotated to APN were over-expressed found by proteomic approaches in LiTOX strain (Tetreau et al., 2012). According to pore formation model, the expression of Bt receptors genes like cadherin should be down-regulated in the resistant insects (Peng et al., 2010; Vachon et al., 2012). However, the current findings were not always consistent with this approach. Based on our observations, along with previous studies, we speculated that APN and cadherin-like protein should have a significant role in Cry1Ah resistance of ACB, and resistance might be associated with the expression of multiple receptors between ACB-AhR and ACB-BtS strains. In this study, the GPI-anchored metabolic pathway was detected in GO annotation, and KEGG pathway analysis and GPI-anchored proteins like ALP were identified as Cry-toxin receptors. ALP expression was under-expressed in *H. virescens* population in a laboratory experiment (Jurat-fuentes et al., 2003). The identification of ALP has been described as Cry-toxin receptors for Cry1Ac (Chen et al., 2015; Jin et al., 2015), Cry11Aa (Fernandez et al., 2006), and Cry4Ba toxins (Moonsom et al., 2007). Generally, the Bt resistance confers changes in the structure of Cry toxin receptors rather than in their expression (Griffitts and Aroian, 2005). These changes in the expression of Cry receptors are likely the result of different genetic mechanisms involving mutations in regulatory regions or genome rearrangements which cause rapid adaptations to new environmental pressure such as an insecticide treatment.

Moreover, GO function and KEGG pathway enrichment were analyzed for DEGs of ACB-AhR and ACB-BtS to find other Cry1Ah-resistance related genes in ACB, as these pathway analyses provide a valuable understanding of the biological process, cellular components and molecular functions of target sites (Ji et al., 2012). The results revealed that the majority of these DEGs were down-regulated in ACB-AhR both from RNA-seq and iTRAQ analyses. These results are in agreement with the Cry1Ab resistance study which showed down-regulation (85.8%) of DEGs in the ACB-AbR strain (Xu et al., 2015). However, the majority of DEGs was significantly up-regulated in a Cry1Ac-resistant strain of *P. xylostella* (Lei et al., 2014). These findings proposed that Cry1Ah-resistance mechanism in ACB can differ from *P. xylostella*, or expression level of up-regulation of genes could be compensated for the loss of other catalytic genes to reduce the fitness costs of Cry toxin resistance. In the present study, expression of mostly genes annotated to GSTs, ATPase, ABCC3, trypsin, and P450 was lower in ACB-AhR. In previous studies, GSTs and P450 genes were reported to confer resistance and were involved in detoxifications of xenobiotic (Xu et al., 2015; Pavlidi et al., 2018), as well as trypsin, which is considered the main proteinase involved in Bt toxin activation and detoxification (Liu et al., 2014). The ABC proteins are membrane bound transporters associated with the movement of solutes across the lipid membranes and have been linked to Bt toxin resistance in the midgut of Cry1Ac and Cry1Ab resistant larvae (Dermauw and Van, 2014; Tabashnik, 2015). In this study, differentially expressed ABC transporters between ACB-BtS and ACB-AhR strains included ABCC1, ABCC2, ABCC3, ABCC4, and Abcc10 and the majority of them were down-regulated. Previously, ABCC2 has been reported to be involved in Cry1Ac resistance in three lepidopterans (Gahan et al., 2010; Baxter et al., 2011). Additionally, eight genes annotated to ABCC2 were detected in Cry1Ac resistance strain of *P. xylostella*, and the majority of them were down-regulated (Lei et al., 2014). Nevertheless, ABCC2 can function as Cry1A toxin receptors (Degen, 2004), and further investigations are required to elucidate the role of genes within Bt resistance mechanisms.

Generally, Cyt toxins identified in the case of previously documented Cry1Ah toxin as receptors of Cry toxins (Perez et al., 2005), possibly contribute to overcoming receptor alterations in ACB-AhR strain. As previously reported in several Bt resistant insects, Cry-toxin resistance might be linked with multiple receptors, and there is a possibility that Cry1Ah resistance is associated with differential expression of Bt toxin receptors between ACB-AhR and ACB-BtS strains. In conclusion, this is the first combination of a study using RNA-Seq and iTRAQ approaches on Cry1Ah-toxin binding, which led to the identification of a longer length of genes in ACB. Besides, Cry1Ah-resistance in ACB is involved in metabolic and catalytic pathways. DEGs and DEPs would be used for further studies on the membrane receptors which are associated with Cry1Ah-resistance and could lead to the analysis of genetic differences between Bt resistant and susceptible strains of ACB.

## Author Contributions

KH and MS designed the experiments. MS and TZ performed the experiments and analyzed the data. ZW and KH provided the insect, reagents, and materials. MS drafted the manuscript. KH and TZ reviewed and edited the manuscript.

## Conflict of Interest Statement

The authors declare that the research was conducted in the absence of any commercial or financial relationships that could be construed as a potential conflict of interest.

## Footnotes

1 https://www.ncbi.nlm.nih.gov/refseq/about/nonredundantproteins/

2 http://www.geneontology.org/

3 http://frodo.wi.mit.edu/primer5/
